# C–H Functionalization/activation in organic synthesis

**DOI:** 10.3762/bjoc.12.224

**Published:** 2016-11-03

**Authors:** Richmond Sarpong

**Affiliations:** 1 Department of Chemistry, University of California, Berkeley, 841A Latimer Hall, Berkeley, CA 94720, USA

**Keywords:** C–H activation, C–H functionalization

The last decade has seen an explosion in research reports in the area of C–H functionalization/activation in organic synthesis. This is not surprising. What is surprising is that it took many in the synthetic organic community this long to fully embrace this exciting and enabling technology. After all, organic compounds mainly consist of a carbon skeleton that bears a large number of hydrogens. It is therefore highly desirable to be able to take advantage of the myriad of C–H groups in organic molecules as functional handles for bond formation, and in some cases, bond-breaking processes.

“Modern” C–H functionalization/activation can trace its roots to the “clarion call” by Bergman and co-workers [1] in a famous *Accounts of Chemical Research* paper in 1995 where the potential for this reaction was discussed. The community has responded and today, there are investigators from all branches of chemistry, chemical biology, and engineering that are pursuing new powerful methods and strategies to achieve C–H functionalization/activation reactions. The growth in popularity of C–H functionalization/activation in organic synthesis can be attributed to the desire to implement more sustainable methods for synthesis and to achieve novel reactivity and selectivity in building molecules. A lot of exciting progress has been made in a short period of time and the stage has been set for even more far-reaching developments in the future.

In this Thematic Series, a collection of 10 contributions from researchers in the area of C–H functionalization from Europe, United States, Japan, China, India, and Brazil is presented. These contributions include full accounts on primary research in the area of C–H functionalization/activation and reviews that focus on aspects of this exciting field. The powerful C_sp_^2^ functionalization of heterocycles provides access to value-added compounds as reported by Itami et al. [2], Chatani et al. [3] and Waser et al. [4]. Alternatively, the C_sp_^2^ functionalization of benzenoids is equally powerful and affords interesting opportunities as described by Lipshutz et al. [5], Bisai et al. [6] and Chen et al. [7]. The emerging power of controlled and selective C_sp_^3^ functionalization is also captured in this series with contributions from Wang et al. [8], May et al. [9], Machado et al. [10] and Dai et al. [11].

I am grateful to all those that have participated in making this Thematic Series, first and foremost, the authors. I am also thankful to the reviewers, who made many constructive suggestions. Overall, it is clear that the field of C–H functionalization/activation in all its varied forms is an exciting area of modern synthetic chemistry and in many ways is revolutionizing synthetic organic chemistry. This Thematic Series provides a view into this burgeoning and exciting world.

Richmond Sarpong

Berkeley, September 2016
